# How data assets influence enterprise persistent innovation: Evidence from China

**DOI:** 10.1371/journal.pone.0331845

**Published:** 2025-09-09

**Authors:** Guohua Cao, Huihui Ye

**Affiliations:** School of Economics and Business Administration, Chongqing University, Chongqing, China; Southwestern University of Finance and Economics, CHINA

## Abstract

This study investigates the impact of data assets on enterprise persistent innovation using panel data from Chinese A-share listed firms from 2011 to 2022. The results indicate that data assets significantly enhance both the inputs and outputs of enterprise persistent innovation, with the findings remaining robust under endogeneity tests. Mediation analysis reveals that data assets influence enterprise persistent innovation through three key channels: process innovation, business innovation, and technological innovation. The development of digital finance positively moderates this relationship across three dimensions of coverage, depth, and digitalization, indicating that digital finance amplifies the persistent innovation value of data assets. Heterogeneity analyses reveal that the persistent innovation input improves more in non-state-owned enterprises, digitally advanced firms, and non-manufacturing sectors, whereas output enhancement is most evident in large enterprises, highly digitalized firms, and organizations with strong absorptive capacity. These findings contribute to a deeper understanding of data-driven persistent innovation and provide valuable insights for policymakers developing data markets, and for firms formulating data strategies aligned with their capabilities.

## 1 Introduction

As a dynamic but enduring process, persistent innovation represents a core strategic capability that enables firms to develop and maintain competitive advantages in hypercompetitive environments [1]. However, the persistence of corporate innovation is often constrained by both endogenous and exogenous factors. Endogenous determinants primarily include knowledge from skilled employees, top management team stability, and intelligent transformation [2–4], while exogenous influences consist of intellectual property protection, external partner cooperation, and business environment conditions [1,5,6]. Yet, the role of data assets, an emerging production factor and critical enterprise resource, in shaping persistent innovation remains underexplored.

From a theoretical perspective, the traditional resource-based view and dynamic capabilities theory primarily focus on tangible and intangible resources (e.g., patents and brands) but they lack a systematic explanation of how data assets contribute to firms’ persistent innovation. This research addresses that gap by proposing that the distinctive attributes of data assets (non-rivalry and reusability) serve as core drivers of enterprise persistent innovation. Furthermore, while dynamic capability theory emphasizes a firm’s ability to adapt to environmental changes, it remains unclear how data assets enhance such capabilities. Accordingly, we establish a data-driven dynamic capability framework to demonstrate how innovation model transformation facilitates enterprise persistent innovation. This research advances the integration of data assets with dynamic capabilities, thereby extending the boundaries of dynamic capability theory.

From an empirical perspective, existing studies have examined the influence of data assets on innovation through two main lenses. One line of research suggests that data assets enhance innovation investment. For example, Zheng et al. argue that data assets improve a firm’s ability to integrate valuable information and increase competitiveness, leading to more intensive innovation inputs [7]. Other studies indicate that data assets substantially boost innovation output, particularly high-quality innovations [8]. Zhang et al. demonstrate that data assets enhance green innovation performance by alleviating financing constraints and improving research and development (R&D) efficiency in environmental technologies [9]. The scale and quality of firms’ data assets are directly correlated with sustainable innovation practices and outcomes [10]. Collectively, these studies highlight the multifaceted role of data assets in driving various dimensions of innovation.

However, this body of research has critical limitations that our study addresses. While existing studies have extensively examined the influence of data assets on discrete innovation investments and outcomes, they systematically overlook the temporal dimension of persistent innovation inputs and outputs, which are key determinants of long-term competitive advantage in the digital economy. The prevailing paradigm fails to capture how data assets create self-reinforcing innovation cycles through continuous accumulation and utilization over time. Our study fills this gap by investigating the mechanisms through which data assets enable enterprise persistent innovation beyond transient effects.

To examine the relationship between data assets and enterprise persistent innovation, we conduct an empirical analysis using from Chinese A-share listed companies from 2011 to 2022. Following the persistent innovation literature [1,4,11], we decompose persistent innovation into persistent innovation input and output. To identify underlying mechanisms, we construct a fixed-effects panel model, conduct a mediation effect test and moderation test. Furthermore, our empirical investigation incorporates multidimensional heterogeneity tests to examine contingent effects.

Our main findings can be summarized as follows. (1) Data assets significantly enhance both the inputs and outputs of enterprise persistent innovation. (2) Data assets influence enterprise persistent innovation through three distinct but interconnected pathways of process, business, and technological innovations. (3) The development of digital finance significantly amplifies the relationship between data assets and enterprise persistent innovation. (4) The enhancement of enterprise persistent innovation input by data assets is more pronounced in non-state-owned enterprises, digitally advanced firms, and non-manufacturers, while the impact on output is most evident in large enterprises, highly digitalized sectors, and firms with strong absorptive capacity.

Based on these findings, this study makes three pivotal theoretical contributions. First, we extend resource-based theory by re-conceptualizing data assets as dynamic capability enablers with unique temporal scalability. Unlike depreciating physical resources, data assets accrue value through use. Our “temporal-innovation-amplification” framework demonstrates how data assets simultaneously enhance innovation inputs and outputs, producing compounding effects on persistent innovation performance. Second, our work bridges resource-based and innovation process theories by revealing an innovation mode mediation mechanism. While traditional innovation research often examines process, business, and technological innovations as static and isolated phenomena, we propose a “data-driven innovation orchestration” framework. This framework shows how data assets dynamically enable the evolution of innovation modes and generate cumulative effects on both innovation inputs and outputs. Accordingly, our model clarifies how data assets contribute to persistent innovation in enterprises. Third, this study expands dynamic capability theory by identifying a novel moderating mechanism. While conventional dynamic capability perspectives emphasize internal adaptation mechanisms (e.g., absorptive capacity, integration capability), we articulate a “digital finance and data co-capability” framework. This framework posits that digital finance systematically enhances the abilities of firms to orchestrate data resources for innovation, thereby positively moderating the relationship between data assets and enterprise persistent innovation.

The remainder of this paper is organized as follows. Section 2 reviews related literature and develops our hypotheses. Section 3 describes variable selection and empirical model specification. Section 4 reports estimation results and robustness checks. Section 5 examines underlying mechanisms. Section 6 presents our heterogeneity analysis. The final section concludes with key findings and limitations.

## 2 Literature review and research hypotheses

### 2.1 Enterprise persistent innovation

Current research on persistent innovation has evolved along two primary theoretical trajectories. The first stream employs green innovation as a proxy for sustainable innovation [9], while the second focuses on endogenous innovation persistence capability [1,4,11]. Although recent attempts have been made to integrate these approaches, their conceptual core remains anchored in environmental innovation [12,13]. This study adopts the second stream framework to examine the impact of data assets, based on the following theoretical considerations. First, persistent innovation better aligns with the endogenous innovation mechanisms of data assets [8]. Fundamentally, data-driven predictive analytics and AI-enabled R&D optimization represent autonomous knowledge accumulation processes, demonstrating theoretical isomorphism with persistent innovation emphasis on continuous technological iteration [11]. In contrast, green innovation predominantly reflects regulatory compliance behavior [14,15]. Second, persistent innovation exhibits superior cross-industry generalizability. The innovation effects of data assets in non-polluting sectors like technology and finance transcend conventional green innovation measurement boundaries [16,17], yet perfectly correspond to persistent innovation. Third, persistent innovation metrics demonstrate enhanced temporal stability. Compared to green innovation indicators that fluctuate with policy cycles such as carbon intensity [16,18–21], persistent innovation more robustly captures the cumulative effects of data assets.

Given these theoretical advantages, persistent innovation has emerged as a prominent research stream in management and economics, yielding substantial theoretical and empirical insights. The current literature presents two representative perspectives on enterprise persistent innovation:

First, from a temporal continuity perspective, persistent innovation is conceptualized as sustained incremental innovation activity over time. Clausen et al. established the foundational concept of innovation persistence in the firm-level innovation literature, showing that prior innovation experience significantly increases the likelihood of subsequent innovation [22]. Triguero and Córcoles employed dynamic probit analysis and demonstrated that innovation inputs and outputs exhibit distinct continuity patterns over time, with particularly strong persistence in inputs [23]. Building on this concept, Xie et al. proposed cyclical growth rates, which are calculated as periodic innovation growth multiplied by current innovation levels, as a superior metric for assessing persistent innovation [11].

Second, from a process-oriented perspective, persistent innovation is viewed as a systematic organizational capability that integrates dynamic processes for sustained performance. Grounded in the dynamic capabilities framework [24], this view emphasizes two key elements: resource reconfiguration and systemic integration. Research shows that firms achieve innovation persistence through continuous organizational learning [25], incremental improvement, and breakthrough initiatives within adaptive innovation systems. These process capabilities are operationalized in two dimensions: innovation outcomes (e.g., patents, new products) and innovation processes (e.g., R&D cycles, knowledge management systems) [8]. Empirical studies confirm that these capabilities support environmental alignment [26] and sustained competitive advantage [22], positioning persistent innovation as a meta-capability of organizational adaptation.

The temporal perspective explains innovation persistence via path dependence, whereas the process perspective reveals the organizational mechanisms that sustain innovation. For empirical analysis, we emphasize temporal continuity based on data availability and alignment with established econometric methods in persistent innovation research [1,4,11].

### 2.2 Data assets and enterprise persistent innovation

The China Academy of Information and Communications Technology defines data assets as “enterprise-controlled data resources capable of generating future economic benefits, recorded in physical or electronic forms (e.g., documents, databases)” [10]. As China has advanced in the digital era, firms have accumulated a wide range of data assets ranging from foundational data repositories to advanced algorithmic systems. The resource-based view (RBV) identifies these assets, including historical transaction records, consumer behavioral data, and proprietary technical data, as valuable due to their scarcity and inimitability, forming a foundation for innovation [27]. However, the RBV offers limited insight into how organizations dynamically leverage and operationalize these data assets. In contrast, the dynamic capabilities theory (DCT) addresses this gap by emphasizing three core processes: (1) the real-time acquisition of data flows, (2) adaptive integration of heterogeneous data sources, and (3) strategic transformation of data into innovative outputs. These processes enable firms to align internal data competencies with changing market conditions, facilitating persistent innovation. Drawing on both the RBV and DCT, we examine how data assets influence enterprise persistent innovation inputs and outputs.

On the input side, data assets enhance firms’ capacity for persistent innovation by improving information integration, optimizing resource allocation, and reducing innovation uncertainty. First, drawing on DCT [24,28], data assets enable firms to integrate valuable information systematically, identify critical innovation resources, and allocate them more effectively. By leveraging integrated internal and external data analytics, firms can more precisely identify high-potential innovation opportunities, enabling more focused and intensive R&D investments [7]. Second, the shared nature of data assets transforms traditional innovation input modes. By using data middleware technologies, firms can share R&D data across departments and projects, thereby avoiding redundant investments [29,30]. This “collect once, use multiple times” approach substantially enhances the marginal returns on innovation inputs. Third, datasets can improve innovation risk management. Advanced data-driven assessment models allow firms to monitor the technical feasibility and market alignment of innovation projects continuously, enabling the dynamic reallocation of R&D investments to mitigate risks and capitalize on emerging opportunities [31]. These findings support the view that data assets function as strategic enterprise resources and reinforce the continuity and resilience of corporate innovation investment cycles. Therefore, we propose the following hypothesis:

**H1a**: *Dat*a assets significantly promote enterprise persistent innovation input.**

Furthermore, data assets enhance enterprise persistent innovation output by accelerating knowledge recombination, expanding innovation boundaries, and amplifying market value. First, based on the knowledge-based view [32], data assets allow firms to deconstruct and recombine existing knowledge elements systematically. Through advanced data mining and machine learning techniques, firms can identify novel combinations of technological components that lead to breakthrough innovations [33]. Second, the scalability of data assets extends innovation boundaries beyond traditional limits. By leveraging large-scale user behavior datasets and real-time feedback loops, firms can develop hyper-personalized products and services that precisely match evolving market demands [34]. Third, data assets increase the market value of innovation. Predictive analytics and dynamic pricing algorithms enable firms to optimize innovation commercialization strategies [35]. These mechanisms demonstrate that data assets act as catalytic converters of innovation output, transforming creative efforts into measurable economic value and sustained competitive advantage. Based on these concepts, we propose the following hypothesis:

**H1b**: *Data assets significantly promote enterprise persistent innovation output.*

### 2.3 Mechanism of data assets in promoting enterprise persistent innovation

Extending the framework of Ning et al.’s [36], this study empirically tests three hypothesized mediation pathways (process, business, and technological innovations) through which data assets support enterprise persistent innovation [37,38]. Drawing on DCT [28] and the RBV [39], we propose an integrated framework in which data assets serve as strategic resources that enhance operational efficiency through agile process reconfiguration, transform value creation model transformation through ecosystem-based business innovation, and support technological capability advancement through data-driven R&D. These mechanisms collectively strengthen persistent innovation input (via resource optimization and risk mitigation) and persistent innovation output (through accelerated commercialization and technological differentiation).

(1) Process innovation

Process innovation enhances operational efficiency, reinforcing enterprise persistent innovation. In the digital era, increasing market complexity and competitive dynamics have exposed the rigidity of traditional processes as critical bottlenecks to organizational adaptation. Data-driven process innovation [34,38,40], promotes dynamic resource allocation [28] and the release of idle resources [40]. This operational enhancement functions through two mechanisms. The flexibility mechanism enables real-time resource reconfiguration, allowing rapid adaptation to environmental changes and accelerating innovation iteration cycles, and fosters the persistence of enterprise innovation output. The redundancy absorption mechanism redeploys freed capacity into strategic innovation initiatives, promoting the persistence of enterprise innovation input. Together, these mechanisms provide the operational foundation for enterprise persistent innovation. Accordingly, we propose the following hypotheses:

**H2a1**: *Data assets indirectly enhance persistent innovation input through process innovation.*

**H2b1**: *Data assets indirectly enhance persistent innovation output through process innovation.*

(2) Business innovation

Business innovation drives paradigm shifts in innovation models by reconfiguring value-creation logic. As industry boundaries blur, homogenized competition in traditional business models intensifies growth pressures [41]. Data-driven business innovation [42–44] achieves this effect through cross-boundary resource integration, ecosystem collaboration, and value proposition reinvention. This model transformation operates via two mechanisms. The value logic reconfiguration mechanism enables continuous revenue streams through servitization, stabilizing long-term innovation investment, and fostering the persistence of enterprise innovation input. It also facilitates market space expansion through multi-agent symbiosis, accelerating innovation commercialization and promoting the persistence of enterprise innovation output. Collectively, these mechanisms support a persistent competitive advantage through innovation. Therefore, we propose the following hypotheses:

**H2a2**: *Data as*sets indirectly enhance persistent innovation input through business innovation.**

**H2b2**: *Data *assets indirectly enhance persistent innovation output through business innovation.**

(3) Technological innovation

Technological innovation serves as a core driver of persistent innovation. In the era of digital transformation, next-generation technologies (e.g., artificial intelligence and blockchain) have accelerated iteration cycles, compelling firms to develop dynamic technological capabilities [45]. Data-driven technological innovation [38,39,46] enabled by advanced algorithm training and digital simulation establishes a capability-enhancement mechanism. This advancement operates through two pathways. The capability-enhancement mechanism enables breakthrough innovations via data-fed artificial intelligence model training, elevating the technical sophistication of enterprise innovation outputs and promoting their persistence. The risk-mitigation mechanism supports high-potential R&D investments through cost-effective digital twins, lowering experimentation barriers and fostering the persistence of enterprise innovation input. Together, these mechanisms form the technological foundation for persistent innovation. Accordingly, we propose the following hypotheses:

**H2a3**: *Data *assets indirectly enhance persistent innovation input through technological innovation.**

**H2b3**: *Data assets indirectly enhance persistent innovation output through technological innovation.*

### 2.4 Moderating mechanism of digital finance

Financial constraints represent a major barrier to sustained R&D investment [47–49], particularly for innovation activities characterized by long gestation periods and high uncertainty. Digital finance has been shown to alleviate these constraints and stimulate innovation effectively [50,51], especially for small and medium-sized enterprises [52,53], by offering alternative lending technologies that bridge the funding gap left by traditional financial institutions.

This concept aligns with financial constraint theory and highlights a paradox, where even data-rich enterprises may face innovation barriers if they lack sufficient funding for essential complementary investments (e.g., artificial intelligence infrastructure, cloud computing). Digital finance addresses this issue through three primary channels: (1) providing essential growth capital for data infrastructure, (2) enabling continuous R&D funding through flexible financial products, and (3) reducing information asymmetries via alternative data analytics. By overcoming these financing bottlenecks, digital finance functions as a key form of institutional innovation, transforming data assets into persistent innovation capacity.

Therefore, we posit that digital finance acts as a critical moderating mechanism that enhances the innovation returns from data assets not only by improving funding access but also by creating a virtuous cycle in which innovation outcomes generate additional data, attracting further investment. Accordingly, we propose the following hypotheses.

**H3a**: *Digital finance positively moderates the relationship between data assets and enterprise persistent innovation input.*

**H3b**: *Di*gital finance positively moderates the relationship between data assets and enterprise persistent innovation output.**

## 3 Research methodology

### 3.1 Data collection and sampling

This study focuses on enterprise persistent innovation using Chinese A-share listed companies as a research sample based on their representativeness in the Chinese context. The initial sample period spans from 2009 to 2022 and was selected to ensure data availability while avoiding distortions from the 2008 global financial crisis. To examine enterprise persistent innovation using equations (1) and (2), which incorporate two-period lags, we require that each firm have at least five consecutive years of observation. This approach balances methodological rigor with sample representativeness, resulting in a final panel dataset covering the period from 2011 to 2022, representing 2,828 unique firms (each with a minimum of three consecutive annual observations) and 19,956 firm–year observations.

A comprehensive panel dataset was compiled from multiple authoritative sources. Financial statement data were obtained from the China Stock Market & Accounting Research (CSMAR) database, while patent information was sourced from the China National Research Data Service. The mediating variables were derived from the Digital Transformation Research Database of Chinese Listed Companies in CSMAR. The digital finance index was drawn from the widely cited Peking University Digital Inclusive Finance Index. This multi-source approach enhances measurement validity through diverse data types.

Several methodological considerations support this research design. First, the 2011–2022 study period provides sufficient temporal distance from the financial crisis while capturing recent innovation patterns in China’s evolving institutional environment. Second, requiring three consecutive years of data addresses potential survivorship bias and enables the proper estimation of lagged effects in dynamic models. Third, the large sample size (19,956 observations) ensures adequate statistical power to detect meaningful relationships. Our research design follows established practices in innovation studies published in leading economic journals while incorporating necessary adaptations for China’s distinct market context and data availability.

### 3.2 Variable specifications

#### 3.2.1 Enterprise persistent innovation.

Earlier studies predominantly used dynamic random-effects probit models to measure persistent innovation by estimating the probability of firms engaging in continued innovation activities based on regression outcomes [2]. Although this approach captures innovation’s dynamic aspects, it does not reflect its cumulative nature. To address this limitation, Triguero and Córcoles proposed an alternative method, which incorporates both the speed and accumulation of innovation [23] and has been widely adopted in subsequent research [1,4,11]. Consistent with these, we quantify enterprise persistent innovation through a bivariate metric that multiplies the cyclical growth rate of innovation activities between adjacent periods by their current mean scale value, measuring both innovation inputs and outputs. This dual-component estimator offers two distinct advantages: (1) the cycle development speed captures persistence, while (2) the average value for innovation activities of two adjacent periods enhances data stability by minimizing stochastic fluctuations. Formally, enterprise persistent innovation for period t is specified as follows:


$$\begin{array}{c} {EPII}_{t}=\frac{I{IN}_{t}+IIN_{t-1}}{IIN_{t-1}+{IIN}_{t-2}}\times \left({IIN}_{t}+IIN_{t-1}\right), \end{array}$$ (1)

$$\begin{array}{c} {EPIO}_{t}=\frac{O{IN}_{t}+OIN_{t-1}}{OIN_{t-1}+{OIN}_{t-2}}\times \left({OIN}_{t}+OIN_{t-1}\right), \end{array}$$ (2)

where *t*, *t − 1*, and *t − 2* represent the current and two preceding years. ${EPII}_{t}$ and ${EPIO}_{t}$ quantify enterprise persistent innovation inputs and outputs, respectively. $I{IN}_{t}$ and $O{IN}_{t}$ denote firms’ R&D expenditures and invention patent applications, respectively.

#### 3.2.2 Data assets.

This study quantifies data assets (*DA*) value as the residual of a firm’s market value after deducting tangible, intangible, and financial assets [54]. Formally, *DA = ln (Market Value − Fixed Assets − Intangible Assets − Financial Assets)*. Market value is calculated as the sum of a firm’s total book liabilities and stock market capitalization. Fixed and “intangible” assets are based on their net values as reported in the balance sheet. Financial assets are aggregated from the following items: trading financial assets, derivative financial assets, short-term investments (net), available-for-sale financial assets (net), held-to-maturity investments (net), long-term debt investments (net), long-term equity investments, investment properties, and entrusted wealth management products. This approach captures the growing role of data in the formation of intangible capital and its disproportionate contribution to firm value.

#### 3.2.3 Mediating variables.

Inspired by Xu et al. [55], we measure three mediating variables—process innovation (*PI*), business innovation (*BI*), and technological innovation (*TI*)—using the China Listed Firm’s Digital Transformation Research Database. The database supplies a curated keyword list and the aggregate frequency of these keywords in firms’ annual reports, excluding the Management Discussion & Analysis (MD&A) sections.

Process innovation is captured by the following keywords: smart manufacturing, intelligent customer service, intelligent marketing, digital marketing, unmanned retail, unmanned factory, mobile payment, third-party payment, NFC payment, human-computer interaction, and social network.

Business innovation is identified through: smart agriculture, intelligent transportation system, smart healthcare, smart home, robo-advisor, smart tourism, smart environmental protection, smart grid, smart energy, internet-based healthcare, digital finance, internet finance, Fintech, financial technology, quantitative finance, open banking, nets union, and internet plus.

Technological innovation is proxied through the keywords: digital twin, metaverse, virtual human, 3D printing, 5G technology, mobile connectivity, mobile internet, industrial internet of things, digital technology, nano-computing, intelligent planning, intelligent optimization, and wearable technology.

To mitigate the severe right skewness of keyword-frequency distributions, we use the natural logarithm of one plus the total keyword count, ln(1 + frequency).

This measurement approach offers two methodological advantages. First, scientific rigor: because data assets generate value through organizational processes that convert data resources into innovation momentum, the database’s advanced text-mining techniques accurately track the three innovation dimensions, providing a solid basis for mediation analysis. Second, statistical robustness: our log-transformed variables align with established text-analysis protocols in finance and accounting [56,57], yield more stable regression coefficients, and alleviate heteroskedasticity concerns. Together, these features ensure that our measures are both theoretically grounded and empirically reliable.

#### 3.2.4 Moderating variables.

The moderating variable in this study is digital finance (*DF*), along with its three primary dimensions: coverage breadth (*DF1*), usage depth (*DF2*), and the digitalization level of digital financial inclusion (*DF3*). Following prior research [50], we adopt the China Digital Financial Inclusion Index published by the Institute of Digital Finance at Peking University as a proxy for digital finance. We use city-level digital finance data, matching the indices of 340 Chinese cities during the study window with micro-level enterprise data based on the registered addresses of listed firms. All digital finance indicators are normalized, with higher values indicating more advanced digital financial development.

#### 3.2.5 Control variables.

We control for the following variables: (1) the natural logarithm of total assets (*Size*), (2) firm age (*Age*), (3) ratio of net profit to total assets (*Roa*), (4) ratio of total liabilities to total assets (*Lev*), (5) natural logarithm of government subsidies (*Subsidy*), (6) growth rate of operating revenue (*Growth*), (7) ratio of cash and cash equivalents to total assets (*Cash*), and (8) equity share of the largest shareholder (*Top1*). We also include year, industry, and province fixed effects. All variables are defined in Table 1.

**Table 1:** Variable definitions.

Variable type	Variable name	Symbol	Definition
Dependent Variables	Enterprise Persistent Innovation Input	*EPII*	See Equation (1)
	Enterprise Persistent Innovation Output	*EPIO*	See Equation (2)
Explanatory Variable	Data Assets	*DA*	Natural logarithm of market value minus fixed assets, intangible assets, and financial assets
Mediating Variables	Process Innovation	*PI*	The natural logarithm of (word frequency + 1)
	Business Innovation	*BI*	The natural logarithm of (word frequency + 1)
	Technological Innovation	*TI*	The natural logarithm of (word frequency + 1)
Moderating Variables	Digital Finance Index	*DF*	The Peking University Digital Financial Inclusion Index (PKU-DFII)
	Breath	*DF1*	Sub-indices of the PKU-DFII
	Depth	*DF2*	Sub-indices of the PKU-DFII
	Digitalization	*DF3*	Sub-indices of the PKU-DFII
Control Variables	Firm Size	*Size*	Ln(Total assets)
	Firm Age	*Age*	Ln(Current year − IPO year + 1)
	Return on Assets	*Roa*	Net profit/ Total assets
	Leverage Ratio	*Lev*	Total liabilities/ Total assets at year-end
	Government Subsidies	*Subsidy*	Ln(Government subsidies received + 1)
	Growth Ability	*Growth*	Year-on-year growth rate of operating revenue
	Cash Flow	*Cash*	Operating cash flow/ Total assets
	Ownership Structure	*Top1*	Shareholding ratio of the largest shareholder

### 3.3 Summary statistics

The sample consists of 19,956 firm–year observations. The statistical characteristics of the key variables are presented in Table 2.

**Table 2:** Descriptive statistics.

Variable	N	Mean	SD	Min	Max
*EPII*	19956	9.759	1.609	2.722	13.829
*EPIO*	19956	4.325	1.592	0	8.237
*DA*	19956	22.725	1.111	20.470	26.160
*PI*	19956	0.318	0.656	0	5.043
*TI*	19956	0.245	0.596	0	5.727
*BI*	19956	0.304	0.659	0	4.875
*DF*	19956	2.982	1.020	0.162	4.607
*DF1*	19956	2.808	1.035	0.020	4.559
*DF2*	19956	3.022	1.068	0.068	5.107
*DF3*	19956	3.485	1.049	0.076	4.671
*Size*	19956	22.388	1.291	19.688	27.216
*Age*	19956	2.255	0.648	0	3.497
*Roa*	19956	0.051	0.041	−0.313	0.203
*Lev*	19956	0.412	0.187	0.052	0.950
*Subsidy*	19956	16.388	2.300	0	22.876
*Growth*	19956	0.310	0.768	−0.632	6.943
*Cash*	19956	0.055	0.067	−0.528	0.839
*Top1*	19956	0.339	0.142	0.090	0.731

(1) Dependent variables: The mean value of *EPII* is 9.759 with a standard deviation (SD) of 1.609, indicating substantial variation across firms. The minimum and maximum values (2.722 and 13.829) suggest significant heterogeneity in firm innovation investment. The average value of *EPIO* is 4.325 (SD = 1.592), reflecting variability in innovation performance. The range (0 to 8.237) confirms that some firms generate no measurable persistent innovation output, whereas others are highly productive.
(2) Core explanatory variable: The mean *DA* is 22.725 with relatively low dispersion (SD = 1.111), suggesting that most firms cluster around the average. However, the range (20.470 to 26.161) indicates that some firms possess significantly more data assets than others.
(3) Mediating variables: The average value of *PI* is 0.318 (SD = 0.656), indicating that many firms report no *PI,* while a few firms exhibit high levels (max = 5.043). The mean value of *TI* is 0.245 (SD = 0.596), exhibiting a right-skewed distribution similar to that of *PI*. The average *BI* is 0.304 (SD = 0.659), suggesting that business model innovation is relatively rare but present in some firms (max = 4.875).
(4) Moderating variables: The mean *DF* index is 2.982 (SD = 1.020), indicating moderate variation in digital financial development across firms. Digital finance breadth (*DF1*; mean = 2.808) and depth (*DF2*; mean = 3.022) exhibit similar distributions. Digital finance digitalization (*DF3*; mean = 3.485) is higher, suggesting that digital financial services are more advanced in terms of digitalization than in breadth or depth.
(5) Control variables. The average firm size (log transformed) is 22.388 (SD = 1.291), confirming a wide range of firm scales. The mean firm age (log transformed) is 2.255 (SD = 0.648), indicating a mix of young and mature firms. The average *Roa* is 5.06%, with some firms experiencing losses (min = −31.27%). The mean *Lev* is 41.19%, suggesting moderate debt levels across firms. The average subsidy (log transformed) is 16.388 with significant variation (SD = 2.300), implying differing levels of government support. The mean growth rate is 31.01% but a high SD (0.768) and maximum value (694.33%) indicate extreme growth disparities. The average cash ratio is 5.45%, with some firms holding substantial liquidity (max = 83.85%). On average, the largest shareholder holds 33.85% of shares with significant variation (SD = 14.17%).

Both *EPII* and *EPIO* exhibit wide dispersion, confirming substantial differences in firms’ innovation capabilities. The high frequency of zero values suggests that many firms do not engage in formal innovation activities, whereas a few are highly innovative. *DF3* (digitalization) is more advanced than *DF1* (breadth) and *DF2* (depth), implying that technological infrastructure may develop faster than service penetration. To ensure broad representation, the sample includes firms of varying sizes, ages, profitability levels, and ownership structures.

To address scale inconsistencies in the dataset, we standardize the variables for subsequent regression analyses. The correlation matrix (Table 3) reveals that no pairwise correlations exceed 0.7, indicating no immediate concerns regarding collinearity. These statistics provide a basis for our regression analyses, ensuring that the empirical models account for the observed variations across firms.

**Table 3:** Correlation matrix of main variables (n = 19956).

	EPII	EPIO	DA	PI	BI	TI	DF
*EPII*	1						
*EPIO*	0.576***	1					
*DA*	0.565***	0.455***	1				
*PI*	0.203***	0.212***	0.097***	1			
*BI*	0.134***	0.117***	0.089***	0.197***	1		
*TI*	0.165***	0.117***	0.089***	0.319***	0.305***	1	
*DF*	0.223***	0.173***	0.174***	0.248***	0.136***	0.173***	1

Correlation coefficients are reported below the diagonal (upper diagonal, left blank). Coefficients significant at the 10%, 5%, and 1% levels are marked as *, **, and ***, respectively. Variance inflation factors for all key variables remained below the conservative threshold of three, suggesting no substantial multicollinearity concerns. The definitions of all variables are shown in Table 1.

### 3.4 Empirical models

To test research hypotheses **H1a** and **H1b** regarding the impact of data assets on enterprise persistent innovation, we utilize equations (3) and (4) to estimate the effects on persistent innovation.


$$\begin{array}{c} EP{II}_{i,t}=\alpha_{0}+\alpha_{1}DA_{i,t}+{\Sigma \alpha }_{n}Controls_{i,t}+Year+Ind+Prov+\varepsilon_{i,t} \end{array}$$ (3)

$$\begin{array}{c} EP{IO}_{i,t}=\beta_{0}+\beta_{1}DA_{i,t}+{\Sigma \beta }_{n}Controls_{i,t}+Year+Ind+Prov+\varepsilon_{i,t} \end{array}$$ (4)

In these models, the subscript i denotes a firm and t denotes the year. $EP{II}_{i,t}$ and $EP{IO}_{i,t}$ represent the persistence of innovation inputs and outputs, respectively. $DA_{i,t}$ denotes data assets. $Controls_{i,t}$ refers to the vector of control variables. We include year (*Year*), industry (*Ind*), and province (*Prov*) fixed effects. $\varepsilon_{i,t}$ is an error term.

## 4 Empirical findings

### 4.1 Baseline regression results

Using a double-log specification, the coefficient on the core variable (data assets) is interpreted as an elasticity. With year, industry, and province fixed effects included, this elasticity estimates the within-year, within-industry, and within-province marginal effect. Table 4 shows that data assets have statistically significant positive effects on both innovation inputs and outputs at the 1% significance level.

**Table 4:** Regression results of data assets and enterprise persistent innovation (input and output).

Variable	(1)	(2)	(3)	(4)
	Enterprise Persistent Innovation Input		Enterprise Persistent Innovation Output	
	*EPII*	*EPII*	*EPIO*	*EPIO*
*DA*	0.411***	0.270***	0.220***	0.204***
	(10.920)	(8.108)	(7.061)	(6.033)
*Size*	0.405***	0.612***	0.383***	0.467***
	(10.310)	(19.155)	(11.694)	(14.237)
*Age*	−0.261***	−0.154***	−0.209***	−0.143***
	(−7.608)	(−4.995)	(−6.256)	(−4.516)
*Roa*	2.162***	1.982***	1.045**	0.511
	(4.958)	(4.985)	(2.256)	(1.134)
*Lev*	−0.337***	0.086	0.220*	0.310***
	(−2.678)	(0.809)	(1.828)	(2.728)
*Subsidy*	0.113***	0.073***	0.115***	0.087***
	(8.854)	(8.006)	(9.665)	(8.728)
*Growth*	−0.0210	0.030*	−0.011	0.072***
	(−1.012)	(1.662)	(−0.462)	(3.838)
*Cash*	0.293	0.294	−0.597**	−0.740***
	(1.243)	(1.459)	(−2.529)	(−3.381)
*Top1*	−0.874***	−0.243**	−0.461***	−0.112
	(−6.074)	(−2.065)	(−2.998)	(−0.791)
*Year FE*	NO	Yes	NO	Yes
*Ind FE*	NO	Yes	NO	Yes
*Prov FE*	NO	Yes	NO	Yes
*N*	19956	19956	19956	19956
*R* ^2^	0.379	0.542	0.265	0.365

Coefficients significant at the 10%, 5%, and 1% levels are marked with *, **, and ***, respectively. Robust standard errors (clustered at the firm level) are reported with *t*-statistics in parentheses. Because all continuous variables are mean-centered in our analyses, the constant term is zero and represents the predicted value at the mean of all independent variables. This applies to all regression tables.

Columns (1) and (3) in Table 4 indicate that a 1% increase in data assets leads to increases of 0.411% in persistent innovation input and 0.220% in persistent innovation output (p < 0.01). Models (2) and (4) indicate that after controlling for industry, year, and province fixed effects, a 1% increase in data assets result in a 0.270% increase in *EPII* and 0.204% increase in *EPIO*. These results suggest that data assets promote enterprise persistent innovation, although with observable efficiency losses in output transformation. These empirical results provide statistically significant support for H1a and H1b. The fixed-effects analysis reveals that industry characteristics, regional disparities, and macroeconomic fluctuations explain approximately 34% and 7% of the effects of data assets on enterprise persistent innovation input and output, respectively, underscoring the importance of institutional environments in realizing the value of data assets.

Furthermore, Table 4 reveals the following insights. (1) A 1% increase in firm size (*Size*) raises innovation input by 0.612% and output by 0.467%, confirming significant economies of scale. (2) The consistently negative coefficients of firm age (*Age*) indicate greater innovation vitality among younger firms. (3) A 1% increase in profitability (*Roa*) enhances persistent innovation input by 1.982%, suggesting that more profitable firms are more willing to invest in innovation. (4) Government subsidies (*Subsidy*) have significantly positive coefficients, indicating their continued effectiveness in supporting corporate innovation. (5) Ownership concentration (*Top1*) has a significant negative effect on sustainable innovation input, where a 1% increase in the largest shareholder’s stake reduces innovation investment by 0.243%, supporting the view that major shareholders’ risk aversion inhibits corporate innovation.

### 4.2 Robustness checks

#### 4.2.1 Instrumental variable (IV) analysis.

We address endogeneity concerns by employing an IV approach, following Lewbel [58]. Specifically, we use the data factor market pilot policy as the instrument (*Treat =* 1 if a city established a data trading platform in year, and *Treat =* 0 otherwise) within a two-stage least squares (2SLS) framework. The results are presented in Table 5. In the first stage, the pilot policy increased data assets by an estimated 9.80%. This effect is statistically significant. The Cragg–Donald Wald F-statistic (131.589) substantially exceeds the Stock–Yogo weak IV critical value (16.380 at the 10% level), mitigating concerns regarding weak instruments. The Kleibergen–Paap rk LM test further supports the instrument’s relevance. In the second stage, Columns (2) and (4) indicate that the instrumental variable have a statistically significant positive effect on both sustainable innovation input (*EPII*) and output (*EPIO*). Endogeneity tests reject the null hypothesis of exogeneity (p < 0.01), supporting the validity of the instrumental variable approach.

**Table 5:** Regression results of instrumental variable method.

Variables	(1)	(2)	(3)	(4)
	First	Second	First	Second
	DA	EPII	DA	EPIO
*DA*	0.098***		0.098***	
	(5.335)		(5.335)	
*IV*		2.111***		3.250***
		(3.951)		(4.482)
*Kleibergen–Paap rk LM*		27.641***		27.641***
*Stock–Yogo weak ID test critical values (10%)*		16.380		16.380
*Cragg–Donald Wald F statistic*		131.589		131.589
*Controls*	Yes	Yes	Yes	Yes
*Year & Ind & Prov FE*	Yes	Yes	Yes	Yes
*N*	19,956	19,956	19,956	19,956

Coefficients significant at the 10%, 5%, and 1% levels are marked with *, **, and ***, respectively. Robust standard errors (clustered at the firm level) are reported with *t*-statistics in parentheses.

#### 4.2.2 Other robustness checks.

To validate our findings further, we conduct the following robustness tests. (1) Dynamic model specification: we augment equations (5) and (6) by including the lagged value (*L.DA*) of the core explanatory variable to capture the dynamic persistence of innovation activities. (2) Pre-pandemic sample: to mitigate the potential confounding effects of COVID-19, we restrict the sample to the pre-2020 period and re-estimate the models. (3) Non-municipality subsample: we exclude firms located in directly controlled municipalities (e.g., Beijing, Shanghai, Tianjin, and Chongqing) to address potential policy heterogeneity.


$$\begin{array}{c} EP{II}_{i,t}=\theta_{0}+\theta_{1}DA_{i,t}+\theta_{2}L.DA_{i,t}+{\Sigma \theta }_{n}Controls_{i,t}+Year+Ind+Prov+\varepsilon_{i,t} \end{array}$$ (5)

$$\begin{array}{c} EP{IO}_{i,t}=\mu_{0}+\mu_{1}DA_{i,t}+\mu_{2}L.DA_{i,t}+{\Sigma \mu }_{n}Controls_{i,t}+Year+Ind+Prov+\varepsilon_{i,t} \end{array}$$ (6)

Column (1) in Table 6 reveals that the current-period coefficient of *DA* is 0.196 (p < 0.01), while the one-period lagged (*L.DA*) coefficient is 0.114 (p* *< 0.01), indicating persistent and gradually accumulating effects of data assets on enterprise persistent innovation input. Column (4) reveals a current coefficient of 0.107 (p* *< 0.01) and lagged coefficient of 0.137 (p* *< 0.01), further confirming the long-term positive impact of data assets on enterprise persistent innovation output. The control variables (e.g., firm size, government subsidies) maintain consistent significance directions relative to the baseline results. Higher *R*^2^ values suggest improved model explanatory power.

**Table 6:** Regression results of other robustness tests.

	(1)	(2)	(3)	(4)	(5)	(6)
	*EPII*	*EPII*	*EPII*	*EPIO*	*EPIO*	*EPIO*
*DA*	0.196***	0.282***	0.273***	0.107***	0.214***	0.238***
	(6.283)	(6.745)	(7.282)	(3.049)	(4.932)	(6.485)
*L. DA*	0.114***			0.137***		
	(3.996)			(4.294)		
*Constant*	−2.538***	−0.081***	−0.004	−3.073***	−0.063***	0.016
	(−3.939)	(−4.096)	(−0.201)	(−4.273)	(−2.858)	(0.785)
*Controls*	Yes	Yes	Yes	Yes	Yes	Yes
*Year FE*	Yes	Yes	Yes	Yes	Yes	Yes
*Ind FE*	Yes	Yes	Yes	Yes	Yes	Yes
*Prov FE*	Yes	Yes	Yes	Yes	Yes	Yes
*N*	17361	13376	16345	17361	13376	16345
*R* ^2^	0.565	0.497	0.520	0.372	0.336	0.333

Coefficients significant at the 10%, 5%, and 1% levels are marked with *, **, and ***, respectively. Robust standard errors (clustered at the firm level) are reported with *t*-statistics in parentheses. Variables are centered on full-sample means (see Table 4). The constant term reflects the shift due to sample restriction.

Column (2) in Table 6 shows that after excluding pandemic-period samples, the coefficient of data assets increases to 0.282 (p* *< 0.01) from the baseline of 0.270, suggesting that pre-pandemic conditions strengthened the positive impact of data assets on persistent innovation input. Meanwhile, the coefficient for innovation output increases from 0.204 to 0.214 (p < 0.01), indicating that data assets exerted a marginally stronger influence on innovation output under pre-pandemic conditions.

Excluding firms headquartered in direct-administered municipalities produces directionally consistent coefficients, while all estimates retain their original significance levels (p < 0.01). This supports the robustness of our core findings to geographic sampling biases.

## 5 Mechanism tests

### 5.1 Testing mediating mechanisms

#### 5.1.1 Modeling approach.

To test the research hypotheses proposed in Section 2.3, we establish the following mediation models to examine the channels through which data assets influence persistent corporate innovation, focusing specifically on the mediating roles of process, business, and technological innovations. The formal mediation models are as follows:


$$\begin{array}{c} M_{i,t}=\beta_{0}+\beta_{1}DA_{i,t}+{\Sigma \beta }_{n}Controls_{i,t}+Year+Ind+Prov+\varepsilon_{i,t}, \end{array}$$ (7)

$$\begin{array}{c} Y_{i,t}=\beta_{0}+\beta_{1}DA_{i,t}+{\Sigma \beta }_{n}Controls_{i,t}+Year+Ind+Prov+\varepsilon_{i,t}, \end{array}$$ (8)

$$\begin{array}{c} Y_{i,t}=\beta_{0}+\beta_{1}DA_{i,t}+\beta_{1}M_{i,t}+{\Sigma \alpha }_{n}Controls_{i,t}+Year+Ind+Prov+\varepsilon_{i,t}. \end{array}$$ (9)

Here, the subscript i denotes a firm and t denotes the year. $M_{i,t}$ denotes the mediators (process, business, and technological innovations). $Y_{i,t}$ represents the dependent variables ($EP{II}_{i,t}$ and $EP{IO}_{i,t}$). $DA_{i,t}$ is the explanatory variable representing data assets. The control variables are consistent with those in the baseline model.

#### 5.1.2 Empirical findings.

The regression results strongly support the hypothesized mediation mechanisms by which data assets enhance both *EPII* and *EPIO*. Our key findings are summarized below.

(1) Process innovation as a mediator

Our mediation analysis confirms that data assets indirectly influence enterprise persistent innovation through process innovation. Data assets significantly enhance process innovation (β = 0.056, p < 0.01). After controlling for data assets, process innovation further contributes to both persistent innovation input (β = 0.183, p < 0.01, Table 7, Column (2)) and output (β = 0.284, p < 0.01, Table 8, Column (2)). The results support H2a1 and H2b1, showing data-driven process innovation enhances persistent innovation through two mechanisms: (1) redundant absorption strengthens persistent innovation input by reallocating idle resources and stabilizing R&D investments, while (2) flexible adaptation boosts persistent innovation output by accelerating cycles through improved resource allocation and reduced iteration time.

**Table 7:** Mediation analysis with *EPII* as the outcome variable.

	(1)	(2)	(3)	(4)	(5)	(6)
	PI	EPII	BI	EPII	TI	EPII
*DA*	0.056***	0.259***	0.067***	0.261***	0.100***	0.248***
	(3.705)	(7.839)	(4.872)	(7.873)	(7.585)	(7.534)
*PI*		0.183***				
		(9.901)				
*BI*				0.130***		
				(6.668)		
*TI*						0.219***
						(9.864)
*Controls*	Yes	Yes	Yes	Yes	Yes	Yes
*Year FE*	Yes	Yes	Yes	Yes	Yes	Yes
*Ind FE*	Yes	Yes	Yes	Yes	Yes	Yes
*Prov FE*	Yes	Yes	Yes	Yes	Yes	Yes
*N*	19956	19956	19956	19956	19956	19956
*R* ^*2*^	0.129	0.547	0.158	0.545	0.190	0.547

Coefficients significant at the 10%, 5%, and 1% levels are marked with *, **, and ***, respectively. Robust standard errors (clustered at the firm level) are reported with *t*-statistics in parentheses. As in Table 4, all variables are mean-centered.

**Table 8:** Mediation analysis with *EPIO* as the outcome variable.

	(1)	(2)	(3)	(4)	(5)	(6)
	PI	EPIO	BI	EPIO	TI	EPIO
*DA*	0.056***	0.188***	0.067***	0.189***	0.100***	0.176***
	(3.705)	(5.723)	(4.872)	(5.670)	(7.585)	(5.326)
*PI*		0.284***				
		(12.634)				
*BI*				0.216***		
				(9.003)		
*TI*						0.274***
						(10.524)
*Controls*	Yes	Yes	Yes	Yes	Yes	Yes
*Year FE*	Yes	Yes	Yes	Yes	Yes	Yes
*Ind FE*	Yes	Yes	Yes	Yes	Yes	Yes
*Prov FE*	Yes	Yes	Yes	Yes	Yes	Yes
*N*	19956	19956	19956	19956	19956	19956
*R* ^*2*^	0.129	0.376	0.158	0.371	0.190	0.373

Coefficients significant at the 10%, 5%, and 1% levels are marked with *, **, and ***, respectively. Robust standard errors (clustered at the firm level) are reported with *t*-statistics in parentheses. As in Table 4, all variables are mean-centered.

(2) Business innovation as a mediator

Data assets significantly promote business innovation (β = 0.067, p < 0.01), which mediates its effect on innovation input (β = 0.130, p < 0.01, Table 7, Column (4)) and output (β = 0.216, p < 0.01, Table 8, Column (4)), confirming H2a2 and H2b2. These findings validate dynamic capabilities theory [43] through two mechanisms: (1) value logic reconfiguration stabilizes persistent innovation input by transforming transactional exchanges into continuous revenue streams through service infusion, while (2) ecosystem symbiosis accelerates persistent innovation output by shortening time-to-market through cross-industry resource integration and real-time demand matching. This dual capability explains how data assets help firms transcend competitive homogeneity while balancing innovation’s time horizons.

(3) Technological innovation as a mediator

Data assets have strongest direct effect on technological innovation (β = 0.100, p < 0.01), which subsequently drives innovation input (β = 0.219, p < 0.01, Table 7, Column (6)) and output (β = 0.274, p < 0.01, Table 8, Column (6)), supporting H2a3 and H2b3. Technological innovation contributes through two main pathways: (1) risk mitigation (e.g., via digital twins) enhances the stability of persistent innovation input by mitigating uncertainties, while (2) capability enhancement (e.g., via AI and data simulation) facilitates persistent innovation output by improving efficiency and expanding technological possibilities.

### 5.2 Testing moderating mechanisms

#### 5.2.1 Modeling approach.

To examine the moderating mechanism of digital finance, we construct the following model:


$$\begin{array}{c} Y_{i,t}=\gamma_{0}+\gamma_{1}DA_{i,t}+\gamma_{2}{MD}_{i,t}+\gamma_{3}DA_{i,t}\times {MD}_{i,t}+{\Sigma \gamma }_{n}Controls_{i,t}\\ \;+\;Year+Ind+Prov+\varepsilon_{i,t}, \end{array}$$ (10)

where the subscript i denotes a firm and t denotes the year. ${MD}_{i,t}$ represents the moderating variables, namely digital finance and its three dimensions: coverage breadth (*DF1*), usage depth (*DF2*), and digitalization level (*DF3*). $Y_{i,t}$ denotes the dependent variables ($EP{II}_{i,t}$ and $EP{IO}_{i,t}$), while $DA_{i,t}$ represents data assets. The control variables are consistent with those used in the baseline model.

#### 5.2.2 Empirical findings.

(1) Digital finance as a moderator

Digital finance plays a significant moderating role in the relationship between data assets and enterprise persistent innovation. This conclusion holds across both dimensions of persistent innovation (*EPII* and *EPIO*). As shown in Columns (1) to (4) in Table 9, the coefficients of the interaction term *DA × DF* are consistently positive and statistically significant at the 1% level, indicating that digital finance strengthens the positive effect of data assets on enterprise persistent innovation. The moderating effect remains robust even after controlling for province fixed effects. These empirical results support hypotheses H3a and H3b, and provide strong evidence for the financial constraint theory, namely that digital finance improves the efficiency with which data assets are transformed into persistent innovation by alleviating financing frictions.

**Table 9:** Moderating mechanism of digital finance.

	(1)	(2)	(3)	(4)
	EPII	EPII	EPIO	EPIO
*DA*	0.270***	0.263***	0.230***	0.197***
	(8.067)	(7.837)	(6.079)	(5.784)
*DF*	0.420***	−0.276	0.275***	−0.440**
	(6.938)	(−1.491)	(4.216)	(−2.387)
*DA × DF*	0.033***	0.030***	0.037***	0.031***
	(2.932)	(2.664)	(3.073)	(2.600)
*Controls*	Yes	Yes	Yes	Yes
*Year & Ind FE*	Yes	Yes	Yes	Yes
*Prov FE*	NO	Yes	NO	Yes
*N*	19956	19956	19956	19956
*R* ^2^	0.530	0.543	0.348	0.366

Coefficients significant at the 10%, 5%, and 1% levels are marked with *, **, and ***, respectively. Robust standard errors (clustered at the firm level) are reported with *t*-statistics in parentheses. As in Table 4, all variables are mean-centered.

(2) Digital finance sub-indicators as moderators

Table 10 presents the regression results when examining the impact of data assets on enterprise persistent innovation (*EPII* and *EPIO*), along with the moderating effects of the three dimensions of digital finance: breadth (*DF1*), depth (*DF2*), and digitalization level (*DF3*). (a) Breadth of digital finance (*DF1*): The interaction term (*DA × DF1*) exhibits a significantly positive moderating effect, indicating that broader coverage of digital financial services strengthens the impact of data assets on enterprise persistent innovation. (b) Depth of digital finance (*DF2*): Again, the interaction term (*DA × DF2*) is significantly positive, indicating that deeper penetration of digital finance in credit and investment activities further enhances the value of data assets.(c) Digitalization Level of digital finance (*DF3*): The moderating effect remains significant but relatively weak, implying that the supporting role of digital infrastructure in the innovation chain may involve certain time lags.

**Table 10:** Moderating mechanism of digital finance sub-indicators.

	(1)	(2)	(3)	(4)	(5)	(6)
	EPII	EPII	EPII	EPIO	EPIO	EPIO
*DA*	0.272***	0.261***	0.265***	0.210***	0.196***	0.200***
	(8.073)	(7.755)	(7.955)	(6.081)	(5.732)	(5.922)
*DF1*	0.386***			0.257***		
	(6.534)			(3.948)		
*DA × DF1*	0.031***			0.038***		
	(2.816)			(3.143)		
*DF2*		−0.058			−0.178*	
		(−0.653)			(−1.926)	
*DA × DF2*		0.030***			0.030**	
		(2.730)			(2.547)	
*DF3*			−0.071			−0.109
			(−1.010)			(−1.510)
*DA ×DF3*			0.031***			0.024**
			(2.875)			(2.233)
*Controls*	Yes	Yes	Yes	Yes	Yes	Yes
*Year FE*	Yes	Yes	Yes	Yes	Yes	Yes
*Ind FE*	Yes	Yes	Yes	Yes	Yes	Yes
*Prov FE*	Yes	Yes	Yes	Yes	Yes	Yes
*N*	19956	19956	19956	19956	19956	19956
*R* ^2^	0.529	0.543	0.543	0.348	0.366	0.365

Coefficients significant at the 10%, 5%, and 1% levels are marked with *, **, and ***, respectively. Robust standard errors (clustered at the firm level) are reported with *t*-statistics in parentheses. As in Table 4, all variables are mean-centered.

In conclusion, data assets are critical drivers of persistent corporate innovation, and digital finance reinforces this effect through multidimensional synergistic mechanisms. The results provide statistically significant support for H3a and H3b, offering empirical evidence for policymakers to refine the digital financial ecosystem.

## 6 Heterogeneity analysis

### 6.1 Enterprise persistent innovation input

#### 6.1.1 Data assets, ownership, and enterprise persistent innovation input.

Research indicates that the share of R&D expenditure by non-SOEs has been steadily increasing, while that of SOEs has declined relatively [59]. Although SOEs receive more subsidies due to policy preferences [49], their R&D investments may suffer from inefficiencies and manipulative practices [60,61]. The development of big data has improved the allocation efficiency of government subsidies and curtailed R&D manipulation by enhancing information transparency and enabling dynamic monitoring, and these effects are more pronounced in non-SOEs [62]. Building on this foundation, this paper proposes that data assets play a significant role in promoting sustained R&D investment in non-SOEs.

To test this hypothesis, following Wang et al. [62], we split the sample into two groups based on state ownership and repeated the analyses for each subsample [62]. Consistent with our conjecture, the coefficient of data assets is larger in the non-SOE subsample, as shown in Columns (1) and (2) in Table 11. Additionally, the coefficient difference between the two subsamples is statistically significant (p = 0.004). The results support our hypothesis.

**Table 11:** Heterogeneous effects of data assets on enterprise persistent innovation input.

	(1)	(2)	(3)	(4)	(5)	(6)
	Ownership		Digital transformation level		Manufacturing dummy	
	SOE	Non-SOE	High	Low	Yes	NO
	EPII	EPII	EPII	EPII	EPII	EPII
*DA*	0.185***	0.299***	0.332***	0.133***	0.195***	0.539***
	(2.916)	(8.738)	(8.727)	(3.015)	(5.820)	(6.967)
*Constant*	−0.272***	0.118***	0.177***	−0.223***	0.194***	−0.487***
	(−6.413)	(5.014)	(9.742)	(−9.403)	(10.465)	(−12.220)
*Difference*	*p *= 0.004***		*p *= 0.000***		*p *= 0.000***	
*Controls*	Yes	Yes	Yes	Yes	Yes	Yes
*Year FE*	Yes	Yes	Yes	Yes	Yes	Yes
*Ind FE*	Yes	Yes	Yes	Yes	Yes	Yes
*Prov FE*	Yes	Yes	Yes	Yes	Yes	Yes
*N*	6705	13251	10487	9469	15184	4772
*R* ^*2*^	0.551	0.561	0.612	0.468	0.585	0.492

Coefficients significant at the 10%, 5%, and 1% levels are marked with *, **, and ***, respectively. Robust standard errors (clustered at the firm level) are reported with t-statistics in parentheses. The significance of cross-group coefficient differences was tested using the Suset method (p < 0.05 indicates significant differences), with corroborative results from the Chow and Fisher tests. Variables are centered on full-sample means (see Table 4). The constant term reflects the shift due to sample restriction.

#### 6.1.2 Data assets, digital transformation level, and enterprise persistent innovation input.

Highly digitalized enterprises have established robust data infrastructures (e.g., cloud computing platforms and AI analytics systems) that enable the more efficient extraction of data value for R&D management [63]. These firms typically adopt agile innovation paradigms, wherein data assets dynamically optimize R&D processes, compress innovation cycles, and generate a self-reinforcing “data assets → R&D → revenue → reinvestment” positive feedback loop. In contrast, low-digitalization firms face technological constraints that limit data utilization efficiency, thereby reducing the marginal contribution of data assets to R&D improvement. Therefore, we hypothesize that the promotional effect of data assets on enterprise persistent innovation input is more significant in highly digitalized firms.

We divided the sample into high- and low-digitalization subsamples using the median digital transformation index. Columns (3) and (4) in Table 11 report the estimation results. The data assets coefficient is larger in the high-digitalization subsample, supporting our assumption.

#### 6.1.3 Data assets, industry attributes, and enterprise persistent innovation input.

In non-manufacturing industries (e.g., services, finance, and ICT), R&D activities rely more on intangible assets such as knowledge, data, and software, and are less constrained by physical production factors (e.g., equipment upgrade cycles). Therefore, we predict that data assets have a stronger positive effect on firms’ persistent innovation investment in non-manufacturing industries. Following Zhao and Ren [64], we divided the sample into manufacturing and non-manufacturing firms and performed regression analyses [64].

Columns (5) and (6) in Table 11 demonstrate that the coefficient of data assets is larger in non-manufacturing firms. These results indicate that the influence of data assets on enterprise persistent innovation input is more prominent in non-manufacturing firms, supporting our assumption.

### 6.2 Enterprise persistent innovation output

#### 6.2.1 Data assets, firm size, and enterprise persistent innovation output.

Given the considerable heterogeneity in the scale and developmental stages of Chinese enterprises, significant disparities exist in resource endowments, innovation capabilities, management models, and risk tolerance [65,66]. These differences lead to substantial variations in the effective utilization of data assets, innovation efficiency, and the speed of innovation iterations across firms. Consequently, we hypothesize that the facilitative effect of data assets on enterprise persistent innovation output exhibits an amplification effect in larger enterprises. Therefore, we divide the sample into large and small subsamples based on the median firm size.

Columns (1) and (2) in Table 12 reveal that large firms exhibit a stronger coefficient of data assets than small firms, with a statistically significant between-group difference. These results strongly support our hypothesis.

**Table 12:** Heterogeneous effects of data assets on enterprise persistent innovation output.

	(1)	(2)	(3)	(4)	(5)	(6)
	Firm Size		Digital Transformation Level		Absorptive Capacity	
	Large	Small	High	Low	High	Low
	EPIO	EPIO	EPIO	EPIO	EPIO	EPIO
DA	0.303***	0.102**	0.205***	0.081*	0.220***	0.103**
	(6.327)	(2.342)	(5.163)	(1.895)	(5.119)	(2.493)
Constant	−0.220***	0.098***	0.252***	−0.311***	0.355***	−0.246***
	(−5.193)	(2.648)	(11.514)	(−12.719)	(14.708)	(−10.823)
*Difference*	*p *= 0.000***		*p *= 0.001***		*p *= 0.002***	
*Controls*	Yes	Yes	Yes	Yes	Yes	Yes
*Year FE*	Yes	Yes	Yes	Yes	Yes	Yes
*Ind FE*	Yes	Yes	Yes	Yes	Yes	Yes
*Prov FE*	Yes	Yes	Yes	Yes	Yes	Yes
*N*	8686	11270	10487	9469	8231	11724
*R* ^2^	0.375	0.188	0.432	0.288	0.425	0.341

Coefficients significant at the 10%, 5%, and 1% levels are marked with *, **, and ***, respectively. Robust standard errors (clustered at the firm level) are reported with t-statistics in parentheses. The significance of cross-group coefficient differences was tested using the Suset method (*p* < 0.05 indicates significant differences) with corroborative results from the Chow and Fisher tests. Variables are centered on full-sample means (see Table 4). The constant term reflects the shift due to sample restriction.

#### 6.2.2 Data assets, digital transformation level, and enterprise persistent innovation output.

Firms with advanced digital capabilities possess both specialized digital talent and cutting-edge infrastructure. By effectively integrating technological competencies, organizational alignment, and ecosystem synergies, these firms systematically incorporate data assets into their innovation processes, thereby achieving significantly greater sustained innovation output. Therefore, we hypothesize that data assets exert a stronger positive effect on sustained innovation performance in highly digitized firms. Following the methodology described in Section 6.1.2, we conduct separate regression analyses for the high- and low-digital-transformation subgroups.

Columns (3) and (4) in Table 12 reveal that high-digitalization firms demonstrate nearly 2.5 times greater data assets elasticity than low-digitalization firms, confirming our hypothesis.

#### 6.2.3 Data assets, absorptive capacity, and enterprise persistent innovation output.

Absorptive capacity facilitates a firm’s ability to recognize the value of external information and effectively deploy it for innovation. As a fundamental organizational capability for assimilating and exploiting external knowledge, absorptive capacity plays a pivotal role in innovation output [8]. Consequently, we hypothesize that the positive impact of data assets on persistent innovation output is significantly amplified in firms with enhanced absorptive capacity. Using the mean R&D personnel ratio as a threshold, we stratify the sample into high- and low-absorptive-capacity cohorts and perform distinct regression analyses for each subsample.

Columns (5) and (6) in Table 12 demonstrate that the coefficient of data assets is significantly larger for firms with high absorptive capacity compared to those with low absorptive capacity, providing strong support for our hypothesis.

## 7 Conclusion

Persistent innovation serves as the primary catalyst for corporate advancement and economic expansion, making the investigation of mechanisms for effective persistent innovation within enterprises a pivotal research imperative. To explain how data assets empower persistent innovation theoretically, we empirically investigated Chinese A-share listed firms (2011–2022) and obtained the following findings.

First, our empirical results demonstrate that data assets significantly enhance enterprise persistent innovation, with findings robust to instrumental variable analysis and lagged model specifications. These findings make dual contributions to the extant literature: (1) they empirically validate Zheng et al. [7] by demonstrating that data assets significantly enhance corporate R&D expenditures, and (2) they extend Zhao and Zhang’s [30] theoretical framework by providing evidence that organizational data assets improve R&D collaboration efficiency through enhanced knowledge sharing and coordination.

Second, data assets promote process, business, and technological innovations, consistent with the findings of Teeping et al. [38]. Notably, technological innovation exhibits the strongest mediating effect, suggesting that the primary value of data assets lies in fostering technological advancement, rather than merely improving operational efficiency or business models.

Third, digital finance positively moderates the innovation effects of data assets through three dimensions: coverage breadth, usage depth, and digitalization level. These findings indicate that well-developed digital finance ecosystems reduce data circulation costs and enhance utilization efficiency, thereby amplifying the innovation value of data assets. These results support the argument that digitally inclusive financial development has dual effects, promoting enterprise persistent innovation [50,51]. They also provide empirical support for the literature documenting digital finance’s innovation-enhancing effect through the mitigation of corporate financing constraints [52,53].

Fourth, heterogeneity analysis revealed that the effect of data assets on persistent innovation input is more pronounced in non-SOEs, highly digitalized firms, and non-manufacturers, whereas their effects on persistent innovation output is stronger among large firms, highly digitalized enterprises, and organizations with strong absorption capacity. These results highlight the need for tailored data assets strategies based on firm-specific characteristics.

Based on these conclusions, this study provides three key theoretical advancements for reshaping the discourse on persistent data-driven innovation.

(1) Dynamic resource perspective. We re-conceptualize data assets as dynamic capability enablers with temporal scalability, thereby challenging the traditional RBV assumption of static, depreciating assets. Empirically, we validate that data utilization drives value accretion and extend the RBV by revealing data’s compounding effects in innovation ecosystems.
(2) Innovation process integration. The proposed “data-driven innovation orchestration” framework bridges resource-based and innovation process theories. Unlike prior studies that treat innovation modes (process, business, and technological) as isolated, we demonstrate how data assets systematically mediate their dynamic evolution by creating cumulative feedback loops between innovation inputs and outputs. This mechanism explains why data-rich firms sustain their innovation outperformance.
(3) Boundary conditions for dynamic capability. We advance DCT by identifying digital finance as a critical moderator. Our “finance–data co-capability” framework reveals that financial digitalization amplifies firms’ ability to harness data for innovation, thereby resolving prior ambiguities regarding contextual enablers of data-driven dynamic capabilities.

Despite the theoretical and empirical contributions of this study, three limitations that provide avenues for future research should be acknowledged.

(1) Sample and contextual constraints. This study focuses exclusively on Chinese A-share listed companies (2011–2022), which may limit the generalizability of our findings to other regions (e.g., developed economies with mature data markets) or firm types (e.g., unlisted SMEs). Future research could validate these mechanisms in cross-country contexts or extend the sample to private enterprises.
(2) Measurement of data assets and innovation. Although this study employs robust proxies for data assets and persistent innovation (inputs/outputs), the inherent complexity of quantifying intangible data assets and their heterogeneous quality (e.g., structured versus unstructured data) may introduce measurement errors. Future research could adopt alternative metrics such as data granularity or industry-specific data valuation models.
(3) Unexplored synergies. This study focuses on data assets as a standalone factor, neglecting their potential interplay with other production factors (e.g., human capital and artificial intelligence adoption). Investigating complementary effects such as how data assets interact with digital workforce skills could yield richer insights into innovation ecosystems. These limitations underscore the need for more nuanced theoretical and methodological advancements in data-driven persistent innovation research. Addressing these gaps could further refine our understanding of the role of data assets in persistent enterprise innovation.
